# In the Blink of an Eye: Reading Mental States From Briefly Presented Eye Regions

**DOI:** 10.1177/2041669520961116

**Published:** 2020-10-05

**Authors:** Gunnar Schmidtmann, Andrew J. Logan, Claus-Christian Carbon, Joshua T. Loong, Ian Gold

**Affiliations:** Eye and Vision Research Group, School of Health Professions, University of Plymouth, Plymouth, UK; Department of Vision Sciences, Glasgow Caledonian University, Glasgow, UK; Department of General Psychology and Methodology, University of Bamberg, Bamberg, Germany; Faculty of Science, University of Waterloo, Waterloo, Ontario, Canada; Department of Philosophy, McGill University, Montreal, Quebec, Canada; Department of Psychiatry, McGill University, Montreal, Quebec, Canada

**Keywords:** facial expressions, emotional states, temporal processing, theory of mind, eyes

## Abstract

Faces provide not only cues to an individual’s identity, age, gender, and ethnicity but also insight into their mental states. The aim was to investigate the temporal aspects of processing of facial expressions of complex mental states for very short presentation times ranging from 12.5 to 100 ms in a four-alternative forced choice paradigm based on Reading the Mind in the Eyes test. Results show that participants are able to recognise very subtle differences between facial expressions; performance is better than chance, even for the shortest presentation time. Importantly, we show for the first time that observers can recognise these expressions based on information contained in the eye region only. These results support the hypothesis that the eye region plays a particularly important role in social interactions and that the expressions in the eyes are a rich source of information about other peoples’ mental states. When asked to what extent the observers guessed during the task, they significantly underestimated their ability to make correct decisions, yet perform better than chance, even for very brief presentation times. These results are particularly relevant in the light of the current COVID-19 pandemic and the associated wearing of face coverings.

Faces are amongst the most complex objects processed by the human visual system and contain a wealth of information (Bruce & Young, 1986). In addition to providing cues to an individual’s identity, age, gender, and ethnicity, faces provide insight into an individual’s emotions, beliefs, and intentions (Leslie, 1995). In other words, faces are the central focus for our ability to attribute mental states to others. As such, facial expressions are an important component of nonverbal communication and provide information which is critical for social interactions (Ekman, 1973). In processing facial expressions, humans recruit an expansive network of brain regions, including the superior temporal sulcus, orbitofrontal cortex, insular cortex, and the amygdala (Haxby et al., 2000; Phillips et al., 1997; Pitcher et al., 2017).

Facial expressions engage rapid visual processing mechanisms; less than 100 ms of exposure is typically sufficient to identify facial expressions of emotion (Calvo & Esteves, 2005; Neath & Itier, 2014). A wealth of evidence, however, points to the view that there are significant differences in the time-course of processing different facial expressions (e.g., happiness and anger) (Milders et al., 2008; Nummenmaa & Calvo, 2015; Palermo & Coltheart, 2004). For example, a number of studies have utilised a simultaneous discrimination paradigm to demonstrate that angry faces are detected more rapidly than either happy or neutral faces (Fox et al., 2000; Hansen & Hansen, 1988; Öhman et al., 2001; Sato & Yoshikawa, 2010; Sawada et al., 2014). In line with this behavioural evidence, it has been reported that the latencies of event-related potentials (ERP) components associated with facial expression processing are significantly shorter for angry, relative to happy, faces (Feldmann-Wüstefeld et al., 2011). It has been proposed that these differences in perceptual latency reflect the ecological salience of particular emotions (Li et al., 2018). Specifically, rapid detection of a facial expression which communicates threat (e.g., an angry face) may be advantageous for survival (Öhman et al., 2001). It should be noted, however, that a number of studies have found no evidence of a detection advantage for angry faces (Becker et al., 2011; Calvo & Nummenmaa, 2008). It has been suggested that these conflicting results can be explained by methodological differences across several studies (Nummenmaa & Calvo, 2015), where detection advantage is rather determined by the perceptual features of an image (e.g., contrast).

The angry-advantage for the detection of emotion notwithstanding, a good deal of evidence points to the conclusion that positive facial expressions (e.g., happiness) are identified more readily than negative expressions (e.g., sadness) (Calvo & Lundqvist, 2008; Leppänen & Hietanen, 2004; Milders et al., 2008; Neath & Itier, 2014; Palermo & Coltheart, 2004). For example, Calvo and Lundqvist (2008) found that happy expressions were identified more accurately and rapidly than faces exhibiting sadness, anger, or fear. This happiness-advantage persists when schematic faces are utilised to control for physical differences between happy and sad faces (Leppänen & Hietanen, 2004). It has been proposed that the happiness-advantage can be explained by the fact that happiness is the only pleasant basic emotion or by the upturned mouth which is a facial manifestation unique to happiness (Nummenmaa & Calvo, 2015). At the other end of the spectrum, it has been reported that fear is recognised more slowly than the other basic emotions (Calvo et al., 2014; Calvo & Lundqvist, 2008; Palermo & Coltheart, 2004; Tracy & Robins, 2008). The evidence for this fear-disadvantage, however, is mixed; other studies have found no difference between the processing of fearful, angry, neutral, and sad facial expressions (Calvo & Nummenmaa, 2009; Milders et al., 2008). It has also been proposed that, rather than being categorised by means of discrete categories, such as happiness or sadness, facial expressions are better described with respect to continuous variables along different dimensions, for example, arousal and valence (Russell, 1994; Schmidtmann et al., 2020, Takehara & Suzuki, 1997). For example, an angry face is high in arousal and negative in valence, while a bored face is low in arousal and average in valence. In support of this latter view in particular, participants’ ratings of facial expressions have been found to be successfully captured by two independent dimensions: valence and arousal (Takehara & Suzuki, 1997). Furthermore, describing facial expressions in terms of dimensions, rather than discrete categories, is more consistent with evidence of differences in perceived facial expression intensity (Hess et al., 1997). More recently, it has been proposed that humans use both categorical and dimensional approaches to process facial expressions (Fujimura et al., 2012; Harris et al., 2012). Nevertheless, most previous investigations of the temporal properties of facial expression processing have focused upon a small number of basic emotions.

As already noted, the majority of previous studies have investigated the time-course of facial expression processing for only a limited number of basic emotions (e.g., happiness, sadness, fear, surprise, disgust, and anger) and for full faces. In this study, we are interested in the temporal dynamics of the perception of facial expressions beyond the basic emotions. Previous literature has shown that the eye region in particular plays an important role in human social interactions and that the expressions in the eyes are a rich source of information about other peoples’ mental states (for recent review, see Grossmann, 2017). To our knowledge, the ability to identify nuanced differences between facial expressions, specifically the eye region alone, which are considerably more similar, in terms of arousal and valence ratings, compared to the basic emotional categories (e.g., happiness or anger) has not been tested before. For example, it has not been established whether humans can reliably identify differences between facial expressions of upset, despondence, and disappointment and whether this information can be extracted from the eye region. Further, the temporal aspects of processing these complex mental states have not been investigated. This study aimed to extend understanding of differences in the time-course of processing facial expressions beyond the basic emotions at short presentation times.

## Methods

### Participants

Thirty individuals (20 women, 2 nonbinary, and 8 men; mean age: 21 years, range: 18–30 years) participated in the study. All participants reported at least 10 years of English fluency and were naïve as to the purpose of the experiment. Participants had normal, or corrected-to-normal, vision. All participants were recruited through a McGill Facebook group. Written informed consent was obtained from each participant. Further details are summarised in Table 1. All experiments were approved by the McGill University Ethics committee (dossier number 51-0714) and were conducted in accordance with the original Declaration of Helsinki.

**Table 1.** Subject Details.

Subject ID	Age	Gender	Race	Religion	Country of origin	Years in Canada	Guess rate
1	23	Male	Asian	Hinduism	India	11	60–70 (65)
2	22	Female	White	None	Canada	22	40
3	21	Female	Multiracial	None	Brazil	0.83	60
4	24	Female	Other	None	Iran	22	40
5	19	Female	White/Asian	Christianity	Canada	19	50
6	30	Male	White	Christianity	Canada	30	40
7	23	Female	White	None	Canada	23	75
8	24	Female	White	Christianity	Canada	24	50
9	20	Male	White	None	Canada	20	65
10	20	Female	Asian	None	China	14	40
11	19	Female	White	Christianity	France	3	30
12	19	Female	Hispanic	None	Colombia	16	90
13	20	Male	White/Hispanic	None	Multicultural	8	55
14	18	Female	Asian	None	Vietnam	1	55
15	21	Male	Asian	None	Vietnam	17	75
16	19	Male	Asian	None	China	1	50
17	24	Female	Asian	None	China	11	60
18	23	Female	Hispanic	Other	Peru	4	50
19	23	Male	Other	Christianity	Peru	3	60
20	22	Female	Asian	Buddhism	China	17	30
21	22	Female	Asian	Christianity	Korea	22	65
22	21	Female	White	None	Russia	9	50
23	20	Male	White	None	Canada	20	40
24	21	Female	Asian	None	Canada	21	35
25	20	Female	Black	Christianity	Canada	20	55
26	22	Non-binary	White	None	United States	25	60
27	21	Non-binary	White/Asian	None	United States	3	75
28	19	Female	Black	Christianity	United States	4	35–40 (37.5)
29	20	Female	White	Judaism	United States	2	15
30	20	Female	White	None	Canada	20	25

### Apparatus

Experiments were performed in a dimly illuminated room. Stimuli were presented, using MATLAB (MATLAB R 2018b, MathWorks) and routines from the Psychtoolbox-3 (Brainard, 1997; Kleiner et al., 2007; Pelli, 1997), on a gamma-corrected Mitsubishi Diamond Pro 2070 CRT monitor with a resolution of 1,280 × 1,024 pixels and a frame rate of 80 Hz (mean luminance: 60 cd/m^2^). The monitor was controlled by a MacBook Air computer (2015, 1.6 GHz). Participants viewed the stimuli at a distance of 55 cm. At this distance, one pixel subtended 0.037° visual angle.

### Stimuli

The stimuli were taken from the McGill Face Database (Schmidtmann et al., 2020). The full McGill Face Database contains colour photographs of 93 different expressions of mental states portrayed by two English-speaking professional actors (one male and one female). For this study, we used the 36 photographs from the McGill Face Database which match the expressions portrayed in the Reading the Mind in the Eyes test (RMET, Baron-Cohen et al., 1997, 2001), which consists of 36 black and white images of eye regions of female (17) and male (19) individuals; taken from magazines. The McGill stimuli were captured for a full-face test of facial expression sensitivity, without any specific focus being placed on the ocular region. The stimuli (18 females and 18 males, see Table 2) were cropped to isolate the eye region, converted to black and white (eight-bit greyscale) and adjusted to match the size of the original RMET stimuli. The resultant stimuli were presented in the centre of a mid-grey background (mean luminance ∼60 cd/m^2^). When viewed at the test distance, the stimuli had a size of 10.4° × 4.0° of visual angle.

**Table 2.** List of Target and Alternative Terms Taken From Baron-Cohen et al. (2001).

	Target term	Alternative 1	Alternative 2	Alternative 3	Actor (sex)
1	Playful	Comforting	Irritated	Bored	Male
2	Upset	Terrified	Arrogant	Annoyed	Female
3	Desire	Joking	Flustered	Convinced	Female
4	Insisting	Joking	Amused	Relaxed	Male
5	Worried	Irritated	Sarcastic	Friendly	Female
6	Fantasising	Aghast	Impatient	Alarmed	Male
7	Uneasy	Apologetic	Friendly	Dispirited	Female
8	Despondent	Relieved	Shy	Excited	Male
9	Preoccupied	Annoyed	Hostile	Horrified	Female
10	Cautious	Insisting	Bored	Aghast	Female
11	Regretful	Terrified	Amused	Flirtatious	Male
12	Sceptical	Indifferent	Embarrassed	Dispirited	Female
13	Anticipating	Decisive	Threatening	Shy	Male
14	Accusing	Irritated	Disappointed	Depressed	Male
15	Contemplative	Flustered	Encouraging	Amused	Male
16	Thoughtful	Irritated	Encouraging	Sympathetic	Female
17	Doubtful	Affectionate	Playful	Aghast	Female
18	Decisive	Amused	Aghast	Bored	Male
19	Tentative	Arrogant	Grateful	Sarcastic	Female
20	Friendly	Dominant	Guilty	Horrified	Male
21	Fantasising	Embarrassed	Confused	Panicked	Female
22	Preoccupied	Grateful	Insisting	Imploring	Male
23	Defiant	Contended	Apologetic	Curious	Male
24	Pensive	Irritated	Excited	Hostile	Female
25	Interested	Panicked	Incredulous	Despondent	Male
26	Hostile	Alarmed	Shy	Anxious	Male
27	Cautious	Joking	Arrogant	Reassuring	Male
28	Interested	Joking	Affectionate	Contended	Female
29	Reflective	Impatient	Aghast	Irritated	Female
30	Flirtatious	Grateful	Hostile	Disappointed	Female
31	Confident	Ashamed	Joking	Dispirited	Male
32	Serious	Ashamed	Bewildered	Alarmed	Female
33	Concerned	Embarrassed	Guilty	Fantasising	Female
34	Distrustful	Aghast	Baffled	Terrified	Female
35	Nervous	Puzzled	Insisting	Contemplative	Male
36	Suspicious	Ashamed	Nervous	Indecisive	Male

The rightmost column shows the sex of the actor representing the facial expression.

The full set of stimuli used in this study can be downloaded here: http://www.gunnar-schmidtmann.com/stimuli-software#TemporalProcessing

The full McGill Face database (Schmidtmann et al., 2020) can be downloaded here: 

http://www.gunnar-schmidtmann.com/stimuli-software#McGillFaceDatabase

### Terms

All 36 target terms from the RMET were tested (Baron-Cohen et al., 2001). The three alternative terms for each target were those established and utilised by Baron-Cohen et al. (2001), shown in their Appendix A. The alternative terms were those identified by Baron-Cohen et al. (2001) during development of the RMET. Note that the terms cautious, fantasising, interested, and preoccupied occur twice in the RMET (referred here to as (a) and (b)). A list of the target terms with the corresponding alternative terms is shown in Table 2.

### Procedure

Participants were initially familiarised with each of the 93 terms which appeared within the test and their corresponding descriptions. Descriptions were extracted from the Glossary of Appendix B in Baron-Cohen et al. (2001).

We employed the same four-alternative forced choice paradigm as that utilised within the original RMET task (Baron-Cohen et al., 2001). The experimental block began with presentation of a mid-grey background. On each trial, participants were shown the eye region of a face portraying an expression for one of eight specific presentation times (12.5, 25, 37.5, 50, 62.5, 75, 87.5, and 100 ms). Within a block, each expression was tested twice at each presentation time (2 × 8 × 36 = 576 trials per block). Expressions and presentation times were presented in a random order using an interleaved design. The stimulus was followed immediately by a mask (presented for 500 ms, 8.8° × 8.8° of visual angle), which comprised random luminance noise. The purpose of the mask was to remove any residual visual transient. Following offset of the mask, participants were presented with four terms (font type: Arial, font colour: white, font size: 60), arranged in a diamond format, on the mid-grey screen. One of the terms (target) described the expression being portrayed by the stimulus. The remaining three terms (distractors) were those established by Baron-Cohen et al. (2001) (Alternative terms in Table 2). The position of the target term (up, down, right, or left) within the diamond configuration was randomly determined on each trial. The participant was asked to choose the term which best described the expression being portrayed by the stimulus. Response was indicated via keyboard press. No feedback was provided. After an experimental block, each observer was asked to estimate the proportion of trials on which they felt that they were guessing the correct term. The guess rates are shown in the rightmost column of Table 1.

## Results

**Figure 1.**
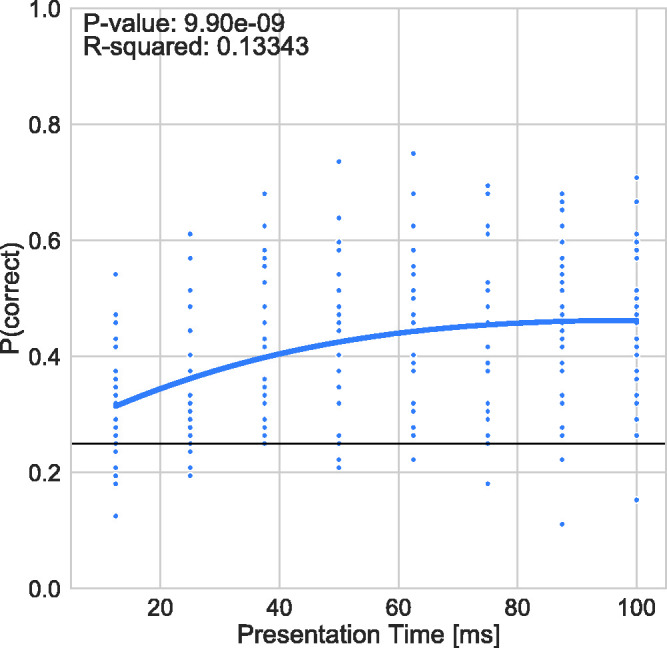
Performance (Proportion Correct) as a Function of Presentation Time. The small circular data points represent mean individual data and the solid blue line shows a third-degree polynomial regression model fit to the data. The black solid line represents the guessing rate of 0.25 P(correct).

Figure 1 shows performance (response accuracy in proportion correct) as a function of presentation time ranging from 12.5 to 100 ms. The small circular data points show the average individual performance for each subject. Performance across observers is shown as the blue solid line, which represents a higher order polynomial regression model fit to the data.

**Figure 2.**
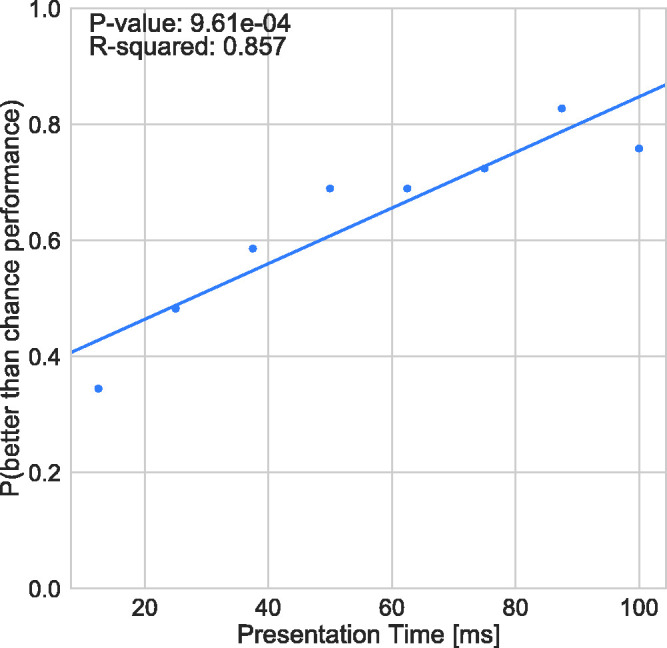
Proportion of Subjects Performing Better Than Chance for Each Presentation Time.

Results show that the average performance increases with increasing presentation time. A third-degree polynomial function was fit to the data (coefficient of determination *R*^2^ and *p* values are shown in Figure 1). Chi-square tests with a Yates correction for continuity (*p* > .05) revealed that on average, participants performed better than chance across all presentation times (Cohen’s *w* for these tests, presentation times less than or equal to 25 ms showed small effect sizes (0.3 > *d* > 0.1), while presentation times greater than 25 ms yielded medium effect sizes (0.5 > *d* > 0.3) (Cohen, 2013). This effect becomes more pronounced as presentation time increases. The proportion of participants exceeding change level increased to over 70% for longer presentation times (see Figure 2).

**Figure 3.**
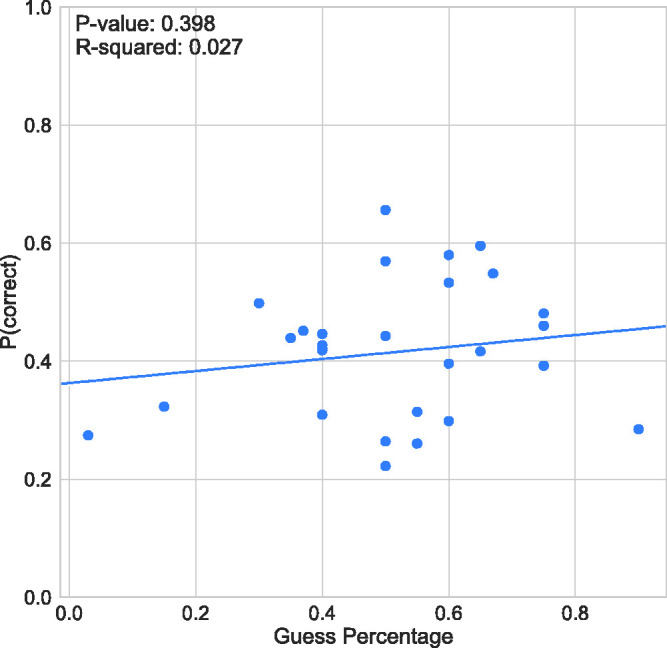
Response Accuracy (Proportion Correct) as a Function of Guess Rate.

The mean guess rate across subjects reported by the observers (see Table 1) is 50.4% (*SD* = 18.5). Figure 3 compares individual estimations of guess rate in comparison to measured accuracy. Linear regression tests found no correlation between these variables *R*^2^ and *p* values are shown in each graph in Figure 3. This analysis suggests that participants were not able to accurately judge the accuracy of their performance.

To investigate whether performance is different for positive or negative terms, the target terms were grouped into positive and negative by consensus of the authors. The following terms were considered to be positive: interested, playful, confident, desire, flirtatious, fantasising, friendly; and the terms upset, worried, doubtful, accusing, nervous, suspicious, hostile, concerned, regretful, despondent, distrustful, and uneasy were identified as negative. The remaining terms were excluded from this analysis because they were considered neutral or ambiguous (e.g., “defiant” and “thoughtful”).

**Figure 4.**
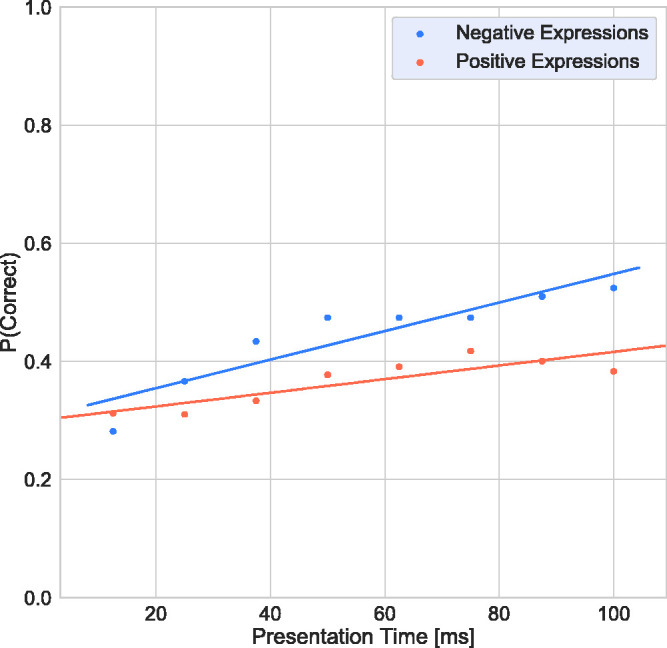
Response Accuracy as a Function of Presentation Time for Negative Terms (Blue) and Positive Terms (Orange).

Figure 4 shows the response accuracy as a function of presentation time. Two-tailed *t* tests (Bonferroni corrected for multiple comparisons), pooled across presentation times, revealed that the overall accuracy of negative terms and positive terms was statistically significant—µ = 0.44, ±0.08 *SD* vs. µ =0.37, ± 0.04 *SD*; *t*(7) = −4.204, *p* = .004; *d* = 0.45.

**Figure 5.**
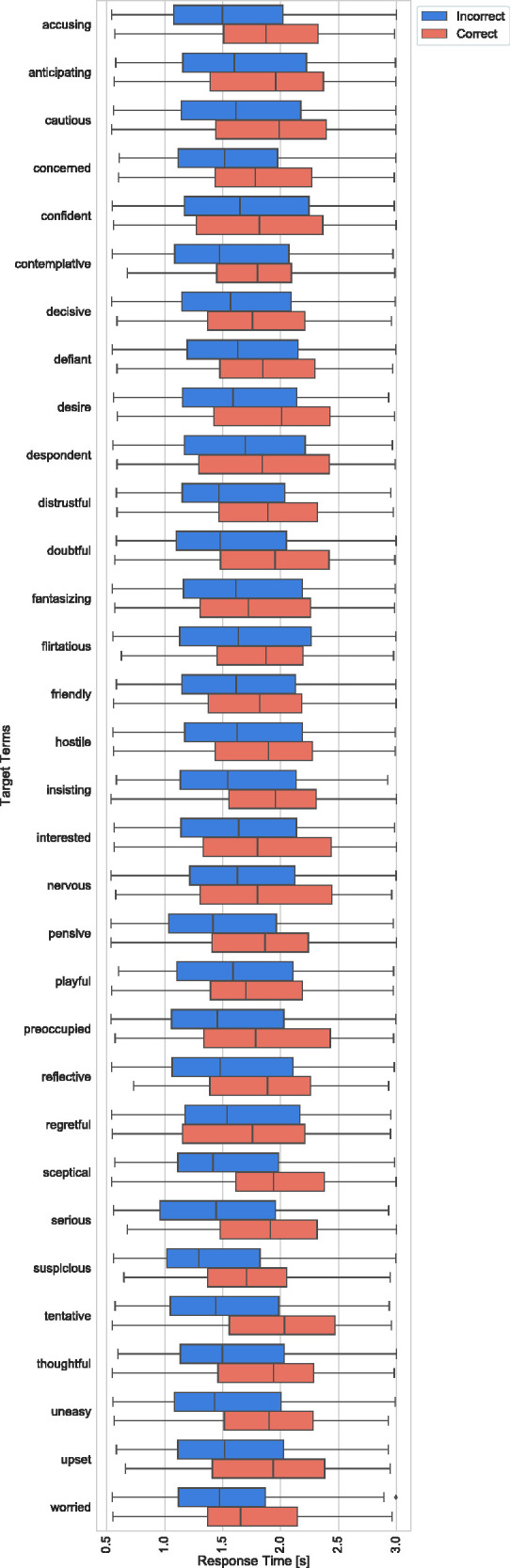
Box-and-Whisker Plots of Response Times (Considering Only Response Times Under 3 s) for Correct (Orange) and Incorrect Decisions (Blue) Across All Participants. The middle line represents the median, the central box displays the IQR, the whiskers being a function of 1.5 the IQR, and the points plotted outside of these whiskers representing the outliers.

Figure 5 shows box-and-whisker plots of response times (considering only response times under 3s) for correct (orange) and incorrect decisions (blue) across all participants. The middle line represents the median, and the central box displays the interquartile range (IQR). The whiskers represent 1.5 times the IQR, and the points plotted outside of these whiskers represent the outliers. Table A1 shows that most response times were statistically significantly shorter for incorrect compared to correct decisions based on two-tailed *t* tests for each target term (Bonferroni corrected for multiple comparisons). In addition, 21 of 30 response times were significantly different (*p* < .05) in mean response time between correct and incorrect decision. In an additional analysis, we analysed the effect size to investigate the magnitude in differences between correct and incorrect response times. The Cohen’s *d* of the observed response times was 0.354. This shows that longer response times had a small magnitude effect (0.5 > *d* > 0.2) on increasing accuracy (Cohen, 2013). Table A2 shows the results for median responses.

**Figure 6.**
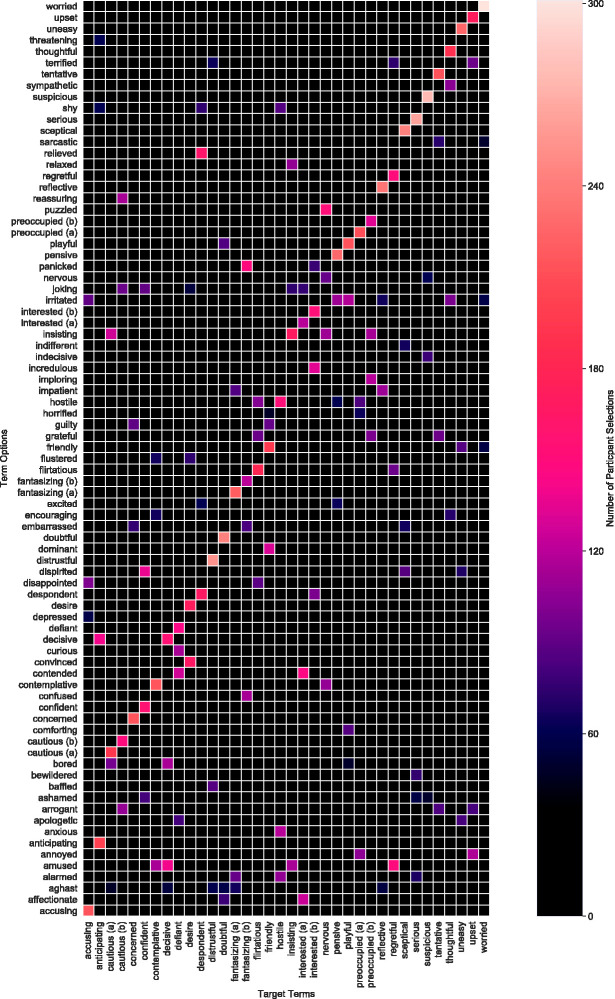
Confusion Matrix. The colour-code refers to the number of selections across all subjects.

Finally, we present the relationship between correct and incorrect responses in a confusion matrix in Figure 6. The colour coding within this matrix represents the number of selections across all participants. The overall diagonal pattern shows that subjects frequently chose the correct term.

## Discussion

The aim of this study was to investigate the temporal aspects of processing of facial (eye region) expressions of complex mental states. Our results show that subjects are able to recognise subtle and fine-grained differences between facial expressions which convey emotions, intentions, and feelings within a fraction of a second—similar results have been revealed before (e.g., Derntl et al., 2009). However, interestingly, humans can recognise these expressions, above chance level, based on information from the eye region only, which underlines the important role of the eye region in social interactions and that the expressions in the eyes are a rich source of information about other peoples’ mental states (Grossmann, 2017). The resolution of visual sensitivity to facial expressions is far superior than might be presumed based on the coarse differences between the Ekman six basic emotions (Ekman, 1992).

In recent years, a number of investigators have pursued the hypothesis that Theory of Mind might be characterised as a dual system or dual process capacity (Apperly & Butterfill, 2009; Frith & Frith, 2008; Meinhardt-Injac et al., 2018). Dual system hypotheses construe a cognitive capacity as subserved by two distinct processes. One—often termed System 1—is typically taken to be unconscious, automatic, and fast, and the other—System 2—conscious, deliberative, and slow (Evans, 2008). Although these properties, among many others, are not necessary features of systems, they are characteristic of them. Our findings provide evidence that mental states can reliably be associated with facial expressions much more rapidly than previously believed, and most importantly, from the eye regions alone. Our results provide some novel support for the existence of a rapid Theory of Mind capacity and, indirectly therefore, for the dual system hypothesis. That facial expressions of complex mental states can be accurately recognised at very brief presentation times might facilitate nonverbal communication and rapid adjustment of one’s approach in response to facial expressions of mental states of another person. Note that our results relate to one specific identity and the extent to which these results can be generalised to other face identities has yet to be determined.

Another surprising finding is that subjects significantly underestimated their ability to make correct decisions at short presentation times. The results shown in Figures 2 and 3 reveal that participants considered themselves to be guessing on a significant proportion of trials, yet they consistently perform better than chance, even for extremely short presentation times. There is a huge body of research showing that emotionally charged stimuli, such as faces with facial expressions, are rapidly and automatically processed (e.g., Almeida et al., 2013; Vuilleumier et al., 2001). Furthermore, it has been shown that responses to emotional stimuli, in particular linked to threat, lead to involuntary decisions (Globisch et al., 1999; Lang et al., 1998; Öhman et al., 1995; Vuilleumier et al., 2001). This might explain the discrepancy between the perceived and actual performance in the task described here. This type of automatic processing of facial expressions of emotional states might have developed to prioritise significant stimuli, presumably those critical for nonverbal communication and social interactions. Here, we can show for the first time that accurate decisions about a person’s emotional state can be extracted in an automatic “pre-attentive” and rapid way from the eye region alone.

**Figure 7.**
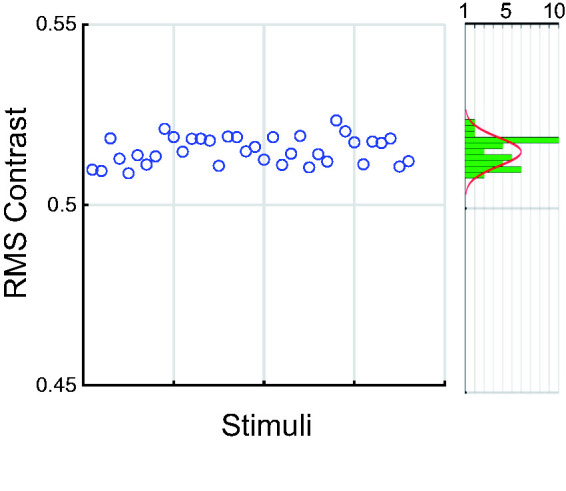
RMS Contrast for the 36 Stimuli. The marginal figure shows the histogram and a normal distribution fit to the RMS contrasts.

As noted in the introduction, the literature on which expressions are more salient—that is, which are more quickly and easily recognised—is mixed. Some have argued that positive expressions like happiness are more easily recognised, while others have argued that it is rather negative expressions like fear or anger that have greater salience (Calvo et al., 2014; Calvo & Lundqvist, 2008; Palermo & Coltheart, 2004; Tracy & Robins, 2008). Our results show that, with increasing presentation time, performance for negative expressions improved much more rapidly than that for positive ones. One might argue that this could be based on image-based aspects of the stimuli used in this study. For instance, Nummenmaa and Calvo (2015) proposed that contrast is a useful cue for rapid identification of expressions. If the stimuli differ across positive and negative groupings in terms of contrast, this could explain the differing results for positive and negative expressions with the two tests. Figure 7 shows root mean square (RMS) contrasts (Pelli & Bex, 2013; Rovamo et al., 1992) for all 36 stimuli. There are insignificant variations across the full range of stimuli including both positive and negative (mean RMS = 0.51, ±0.004 *SD*). Image properties are therefore very unlikely to explain the observed results. It is, however, important to emphasise that this analysis does not provide any information about the contrast distribution (more pronounced local features) which could be responsible for differences.

It is noteworthy that face expression identification accuracy saturates on average around 55% (see Figure 1). Restriction of available information to the eye region may partly explain this limitation of performance. It is well established that the eyes make a disproportionate contribution to the identification of emotional facial expressions (Baron-Cohen, 1997; Grossmann, 2017; Jack et al., 2012). Previous studies, however, have indicated that other face features (e.g., nose, mouth) also communicate information which facilitates interpretation of facial expressions (Baron-Cohen, 1997; Eisenbarth & Alpers, 2011; Yuki et al., 2007). This suggests that an improvement in accuracy may be achieved if the stimuli were adapted to include more face information.

In summary, we can show for the very first time that humans can recognise facial expressions of complex mental states, above chance level, within a fraction of a second, based on information from the eye region only, which underlines the important role of the eye region in social interactions, and that the expressions in the eyes are a rich source of information about other peoples’ mental states. In other words, the eyes really are “. . . windows into other minds . . .” (Grossmann, 2017). The salience of the eye region for inferring an individual's emotional state may be particularly beneficial in situations where information from other features, such as the mouth, is unavailable. This is of particular relevant in the light of the current COVID-19 pandemic and the associated wearing of face coverings (see also Carbon, 2020).

## Appendix

**Table A1.** List of Target Term Response Time (Mean), Two-Tailed *t*-Test Results (Bonferroni Corrected).

Target term	Mean response time correct	Mean response time incorrect	Degrees of freedom	*t* statistics	*p* value
Accusing	1.88	1.59	377	−4.72	0.0001
Anticipating	1.87	1.66	368	−3.26	0.0394
Cautious	1.90	1.67	755	−4.70	0.0001
Concerned	1.82	1.58	403	−4.46	0.0003
Confident	1.82	1.72	347	−1.32	1.0000
Contemplative	1.81	1.59	407	−3.85	0.0044
Decisive	1.78	1.65	373	−2.08	1.0000
Defiant	1.85	1.68	377	−2.40	0.5443
Desire	1.93	1.67	373	−3.94	0.0034
Despondent	1.84	1.69	353	−2.06	1.0000
Distrustful	1.88	1.59	372	−4.75	0.0001
Doubtful	1.91	1.61	391	−4.79	0.0001
Fantasising	1.78	1.69	766	−1.88	1.0000
Flirtatious	1.84	1.70	371	−2.17	0.9835
Friendly	1.81	1.67	390	−2.26	0.7722
Hostile	1.86	1.69	376	−2.82	0.1644
Insisting	1.90	1.64	381	−4.25	0.0009
Interested	1.85	1.68	707	−3.20	0.0475
Nervous	1.83	1.66	394	−2.01	1.0000
Pensive	1.86	1.52	389	−5.76	0.0000
Playful	1.77	1.62	390	−2.40	0.5339
Preoccupied	1.83	1.58	722	−5.01	0.0000
Reflective	1.86	1.62	381	−3.74	0.0067
Regretful	1.71	1.66	356	−0.69	1.0000
Sceptical	1.95	1.57	382	−6.46	0.0000
Serious	1.91	1.53	402	−6.18	0.0000
Suspicious	1.75	1.45	422	−5.74	0.0000
Tentative	1.98	1.56	352	−6.45	0.0000
Thoughtful	1.88	1.60	350	−4.32	0.0007
Uneasy	1.88	1.57	395	−5.33	0.0000
Upset	1.90	1.59	389	−4.81	0.0001
Worried	1.74	1.50	428	−4.71	0.0001

**Table A2.** List of Target Term Response Time (Median), Moody’s Median (Bonferroni Corrected).

Target term	Median response time correct	Median response time incorrect	Degrees of freedom	*t* statistics	*p* value
Accusing	1.88	1.59	377	21.96	.0049
Anticipating	1.87	1.66	368	14.34	.0026
Cautious	1.90	1.67	755	18.88	1.0000
Concerned	1.82	1.58	403	19.64	.0000
Confident	1.82	1.72	347	1.42	.0000
Contemplative	1.81	1.59	407	14.59	.0000
Decisive	1.78	1.65	373	1.61	1.0000
Defiant	1.85	1.68	377	7.88	.0719
Desire	1.93	1.67	373	10.07	.0003
Despondent	1.84	1.69	353	0.87	.0034
Distrustful	1.88	1.59	372	18.92	.0004
Doubtful	1.91	1.61	391	22.05	.2041
Fantasising	1.78	1.69	766	1.42	1.0000
Flirtatious	1.84	1.70	371	3.47	.0000
Friendly	1.81	1.67	390	9.34	1.0000
Hostile	1.86	1.69	376	7.44	.0005
Insisting	1.90	1.64	381	15.02	.0001
Interested	1.85	1.68	707	3.07	1.0000
Nervous	1.83	1.66	394	2.28	.0092
Pensive	1.86	1.52	389	29.30	.1596
Playful	1.77	1.62	390	3.33	1.0000
Preoccupied	1.83	1.58	722	18.73	1.0000
Reflective	1.86	1.62	381	13.16	.0000
Regretful	1.71	1.66	356	1.57	.0000
Sceptical	1.95	1.57	382	42.76	.0043
Serious	1.91	1.53	402	25.14	.0004
Suspicious	1.75	1.45	422	23.77	.0482
Tentative	1.98	1.56	352	38.61	.0000
Thoughtful	1.88	1.60	350	25.89	.0001
Uneasy	1.88	1.57	395	29.95	1.0000
Upset	1.90	1.59	389	15.53	1.0000
Worried	1.74	1.50	428	4.11	1.0000
